# What Do MBA Program in Southeast Asia Scholars Propose for Future COVID-19 Research in Academic Publications? A Topic Analysis Based on Autoencoder

**DOI:** 10.1177/21582440231182060

**Published:** 2023-06-19

**Authors:** Lihuan Guo, Wei Wang, Yenchun Jim Wu

**Affiliations:** 1 Tan Siu Lin Business School, Quanzhou Normal University, Quanzhou, Fujian, China; 2 Cloud Computing, IoT, E-commerce Intelligence Engineering Research Center of Colleges and universities in Fujian Province, Quanzhou Normal University, Quanzhou, Fujian, China; 3 College of Business Administration, Huaqiao University, Quanzhou, Fujian, China; 4 MBA Program in Southeast Asia, National Taipei University of Education, Taipei, Taiwan; 5 Graduate Institute of Global Business and Strategy, National Taiwan Normal University, Taipei, Taiwan

**Keywords:** autoencoder, COVID-19, deep learning, text mining, topic model

## Abstract

To analyze the directions for future research suggested and to project future research plans, we extract relevant text from these publications with respect to COVID-19-related research based on 54,136 relevant academic journals published from the initial outbreak of COVID-19 in January 2020 until December 2020. First, we extract and preprocess the corpus and then determine that, according to the Elbow method, the optimal number of clusters is 7. Then, we construct a text clustering model based on an autoencoder, with the support of an artificial neural network. Distance measurements, such as correlation, cosine, Braycurtis, and Jaccard are compared, and the clustering results are evaluated with normal mutual information. The results show that cosine similarity has the best effect on clustering of COVID-19-related documents. A topic model analysis shows that the directions of future research can mainly be grouped into the following seven categories: infectivity testing, genome analysis, vaccine testing, diagnosis and infection characteristics, pandemic management, nursing care, and clinical testing. Among them, the topics of pandemic management, diagnosis and infection characteristics, and clinical testing trended upward in proportion to future directions. The topic of vaccine testing remains steady over the observation window, whereas other topics (infectivity testing, genome analysis, and nursing care) slowly trended downward. Among all the topics, medical research comprises 80%, and about 20% of the topics are related to public management, government functions, and economic development. This study enriches our scientific understanding of COVID-19 and helps us to effectively predict future scientific research output on COVID-19.

## Introduction

Since the outbreak and large-scale spread of COVID-19 in January 2020, the academic community has carried out extensive research, and many academic papers have been published. These publications are in various fields: medicine, immunology, biology, chemistry, infectious diseases, management, and data analysis (Bikdeli et al., 2020; Sunar et al., 2022). These research results across multiple fields explore COVID-19 from various perspectives. The research topics and contents across these research findings vary. Due to the differentiation among research topics and research methods, it is difficult to analyze the numerous research results using a fixed mode. However, in general, the articles include is a section or a separate paragraph called “Limitations and Directions for Future Research.” This section describes the deficiencies of the study and proposes possible directions for future research. This portion of the text usually follows a relatively fixed format, with a limited length. Thus, it is feasible to analyze the future directions for COVID-19 research.

Topic extraction is a common method used in text analysis to helps us understand lengthy content or high numbers of documents (Maree, 2020). Text topic extraction is a clustering algorithm that divides semantically similar documents into a cluster, maximizes the distance between clusters, and minimizes the distance between the nodes in the cluster (Curiskis et al., 2020). Many algorithms have been proposed for addressing text clusters, such as latent dirichlet allocation (LDA) and hierarchical clustering. Among these algorithms, the most widely adopted is the unsupervised algorithm (Eslami et al., 2020), which does not need to specify the topic and number of clusters in advance. However, the disadvantages of these unsupervised algorithms are obvious, which may lead to different results with the same corpus (Alam & Paul, 2020).

In the relevant research, scholars use the topic modeling and evolution method to cluster the COVID-19 publications from Semantic Scholar (Liu et al., 2021; Ostaszewski et al., 2020). However, the existing research often uses the full text as corpus, which is a summary of publicly available COVID-19 findings; however, they do not project directions for future research on COVID-19 or analyze plans for future research. To analyze the future directions of scholarly work, in this study, using real data from COVID-19-related publications, we employ text mining to identify the directions for future research in the publications, analyze the future directions in research on the COVID-19, and detect their evolution over time (Arshad et al., 2022; Yuan & Wang, 2020). This study enriches future research on COVID-19 from a theoretical perspective. Practically, it provides a reference for scholars who are selecting appropriate research directions, predicting and managing the possible output of future research, and driving the prevention and treatment of COVID-19.

The rest of the paper is organized as follows. Section 2 reviews the literature and proposes research questions. Section 3 presents the research data and research model. The results and analyses are presented in Section 4. Finally, Section 5 concludes with some discussion and future directions.

## Literature Review and Research Questions

In this section, we review the related literature and then, based on the literature review, describe the research gap and propose our research questions.

### Literature Review

In 2020, the outbreak of COVID-19 attracted worldwide attention and caused many serious social problems. Social life was limited in some regions, and some industries were fatally damaged (Sohrabi et al., 2020). In view of the serious impact of the COVID-19 pandemic, scholars have conducted large-scale research from various perspectives. Many of these studies took a medical point of view (Li et al., 2020), and others viewed the crisis in terms of pandemic management, social governance, and economic impacts (Lytras et al., 2020; Weible et al., 2020). The results of their research can be divided into two main groups. The first is the medical perspective: in these studies on COVID-19, the main topics are analyses of viral infection (Tian et al., 2020), vaccine testing (Lurie et al., 2020), and genome analysis (Li et al., 2020). The second is the perspective of management and the social impact of COVID-19, which widely confirms the negative impact of COVID-19 on the economy and society (Ali & Alharbi, 2020).

However, these research results summarize past studies, which might not be the same as plans for research in the future and will be the basis for guiding next steps and investment for the public, law enforcement, regulatory agencies, and researchers. It is difficult to systematically predict future directions by referring to current research. The same applies to research methods: it is difficult to use experimental methods used in medical research today to predict the methods employed in research tomorrow. However, we can gather a corpus of text in current papers to discern patterns (Arman et al., 2014), and this method also applies to studies on COVID-19. To identify the patterns in the narratives, we can use text mining, which is widely adopted in many fields. For example, by combining time factors and topic models, some researchers find that the research topic of COVID-19 has evolved over time (Liu et al., 2021). Other studies include artificial intelligence to analyze medical images related to COVID-19 (Albahri et al., 2020). The common element in this stream of research is that it sorts and summarizes existing research results, but lacks an effective analysis and prediction of possible directions for future research.

Predicting the trends in research on COVID-19 involves two main concerns: the technology for performing text analysis and formation of the corpus. First, based on the technical method of text mining, topic analysis is an effective method for revealing knowledge (M.-Y. Chen et al., 2020; Park, 2022). Another influential method is LDA, one of the most commonly used algorithms in topic analysis (Blei et al., 2003). LDA is a Bayesian probability model, whose structure has three tiers: word, topic, and document. In the so-called generative model, every word in a document is identified through the process of selecting a topic with a certain probability and selecting a word from this topic with a certain probability (Ahmed et al., 2022). Both the document-to-topic distribution and the topic-to-word distribution obey a polynomial distribution (B. Xu et al., 2020). Many improved LDA models have been used in papers on COVID-19. For example, researchers have identified the evolution in public opinion on COVID-19. The results show that public opinion has evolved (Zhuang et al., 2021).

Second, most existing research on the selection of a corpus regarding the COVID-19 pandemic uses online text for the corpus from two main sources. The first is online text created by the public—for example, public attitudes toward public policy expressed in tweets (Xue et al., 2020). The second comprises scientific publications—for example, the current progress in research on COVID-19 can be effectively identified with bibliometric and scientometric analysis of journal articles (Tran et al., 2020).

Autoencoder is an unsupervised learning algorithm with an artificial neural network used in the implementation of text analysis (Kwon et al., 2020; L. Zhang et al., 2020). In large-scale learning, such as text mining, high dimensionality leads to low computational efficiency. Many machine learning (ML) algorithms aim to reduce the dimension or “compress” the data to reduce the ML burden (Meng et al., 2020). The purpose of an autoencoder is to reduce the dimension through a neural network and reduce the loss as much as possible in the process of dimension reduction (T. Chen et al., 2019). Autoencoders have been applied in many fields with good results (Ashfahani et al., 2020), which shows the potential for using them with studies on COVID-19.

### Literature Gap and Research Questions

At present, our understanding of COVID-19 is growing rapidly, and research has yielded many results, but the research topics are scattered. A summary of the current research results demonstrates some shortcomings: First, many studies on COVID-19 focus on immunity, virus detection, and vaccine testing, but few studies focus on potential topics of future research—that is, they only explore the urgent problems in the present but lack a deep analysis of the directions for research going forward. Potential topics could determine future directions not just for research but for investment, which has some theoretical and practical value. Second, in text mining, some scholars have used autoencoders to reduce the dimension of the original data (Dong et al., 2020; Lytras et al., 2019), but did not examine academic journal articles. Third, although it has been over 3 years since the first outbreaks of COVID-19, scholars have not conducted exploration on the evolution of studies on the virus. It is unclear whether scholars’ plans for future research have changed over this period. Hence, we propose the following research goals:

- The first goal is to extract the future directions in research on the COVID-19 from a massive number of published papers, to use various distance measurements to cluster this text, and to analyze topics proposed for future research on COVID-19.
- The second goal is to identify evolution in the topics proposed. That is, we attempt to determine the dynamic changes in the topics based on the topic model and time factor, which will guide researchers in carrying out research effectively in the future.

## Research Model and Research Data

In this section, we propose the research framework. Then, based on our research process, we introduce the key research steps, including the extraction of future directions, data preprocessing, text processing, topic analysis, and research data.

### Research Framework

**Figure 1.**
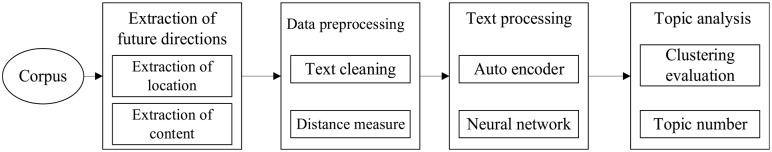
Research framework.

This study begins with extraction of future directions from the papers (Ostaszewski et al., 2020) and preprocessing of the data and processing of the text. Finally, we evaluate the clustering results using topic analysis. Figure 1 illustrates the research framework.

### Extraction of Future Directions

A paper contains various sections, including the title, abstract, introduction, and conclusion, which often comprises a section with future directions. We extract the content of this future directions section, which is generally at the end of the paper, making facilitates extracting the text.

To improve the accuracy of content extraction, we take the following two steps. In the first step, we extract the future directions text based on the text location, which as mentioned earlier is usually the last paragraph. However, a few papers lack this kind of future directions, so we must evaluate the content semantically. So, in the second step, we define some keywords, such as “prospective” and “future directions.” Only paragraphs with these keywords are considered as containing future directions. To confirm the accuracy of our text extraction, we select 200 random samples of future directions and label them manually. Then, we calculate the precision, recall, and F1, and the results are 94%, 91%, and 92%, respectively. Therefore, our extraction of future directions’ content is considered highly accurate.

### Data Preprocessing

#### Text Cleaning

Using the following data preprocessing steps, we process the corpus to obtain standard text data with Python 3.5.

1. Data processing on Meta and JavaScript Object Notation (JSON): the original data form a metafile and corresponding JSON files. The metarecords and JSON files have a lot of duplication. We import the raw data from the metafile, delete the duplicate records, and import the contents.
2. Data cleaning: in this step, we omit several types of papers: (1) we delete papers in languages other than English. Papers in German comprise approximately 0.01% of the papers, and others are in Spanish, French, Dutch, and Chinese. However, publications in languages other than English make up less than 1% of the total, hence, to simplify the data processing, we omit all non-English-language papers. (2) Papers published before 2020 are deleted. The raw data set includes a low number of papers published before 2020, but they differ from the papers on COVID-19 research in 2020. (3) Papers that provide only the author names, titles, and abstracts but lack full-text content are deleted, as our research interest is future directions. (4) We delete data that is duplicated in the raw corpus.
3. Text normalization: This step includes the deletion of punctuation, conversion to lower case, and removal of stop words. To do so, we employ the *stop_words* module in the *spacy* package. In addition, we customize additional stop words for the corpus, such as *paper*, *study*, *research*, *future*, and *plan*. Then, we use the *en_core_sci_lg* module in the *scispacy* package to parse the text. *Scispacy* is a specialized biomedical natural language processing package (Y. Zhang et al., 2021).
4. Vectorization: in this step, the processed text is vectorized into a structure that is convenient for the following processes. The rows in the vector represent the documents, and the columns represent the words. We use the term frequency–inverse document frequency (TF-IDF) algorithm to reduce the dimension and set the number of features at 5,000.
5. Distance calculation: In this step, we calculate the distance between words through correlation, cosine, Jaccard, and Braycurtis. The basic principle is that when two words frequently coexist, they have greater similarity.

**Figure 2.**
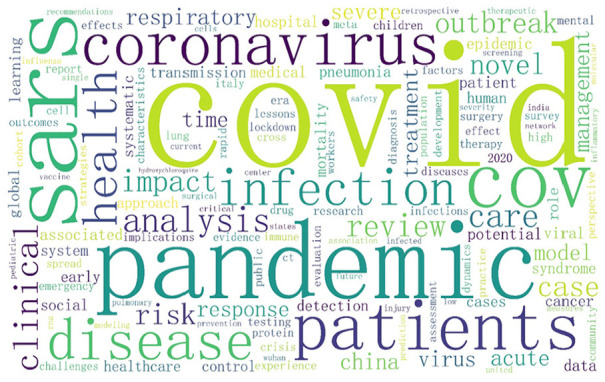
Word cloud.

Figure 2 shows the word cloud, which is produced by the *wordcloud* package in Python. Many words frequently used, as the figure shows, are medical terms, such as *coronavirus*, *sars*, *pandemic*, and *disease*. However, some of them are related to public management, such as *management*, *healthcare*, and *community*. The presence of keywords from multiple fields indicates the dispersion of COVID-19 pandemic research.

#### Distance Measure

Correlation, cosine, Jaccard, and Braycurtis are used to measure the distance between words. Equations (1) to (4) show formulas for these four kinds of similarity, where *s* and *t* indicate any two words, $x_{s}$ and $x_{t}$ are vectors composed of words *s* and *t*, *n* is the length of the vector, and *k* is the iteration factor.

(1) $${\mathrm{Cosine}}_{s,\;t}=\frac{x_{s}x_{t}}{∥x_{s}∥\cdot ∥x_{t}∥}=1-\frac{\sum_{k=1}^{n}x_{\mathrm{sk}}x_{\mathrm{tk}}}{\sqrt{\sum_{k=1}^{n}x_{\mathrm{sk}}^{2}}\sqrt{\sum_{k=1}^{n}x_{\mathrm{tk}}^{2}}}$$

(2) $$\begin{array}{c} {\mathrm{Correlation}}_{s,\;t}=\\ 1-\frac{\left(x_{s}-\bar{x_{s}}\right){\left(x_{t}-\bar{x_{t}}\right)}^{\prime }}{\sqrt{\left(x_{s}-\bar{x_{s}}\right)\cdot {\left(x_{s}-\bar{x_{s}}\right)}^{\prime }}\cdot \sqrt{\left(x_{t}-\bar{x_{t}}\right)\cdot {\left(x_{t}-\bar{x_{t}}\right)}^{\prime }}},\\ \bar{x_{s}}=\frac{1}{n}\sum_{i}x_{\mathrm{si}}\;\mathrm{and}\;,\bar{x_{t}}=\frac{1}{n}\sum_{i}x_{\mathrm{ti}} \end{array}$$

(3) $${\mathrm{Jaccard}}_{s,\;t}=\left(\frac{\#\left[(x_{s_{j}}\neq x_{t_{j}})\cap \left((x_{s_{j}}\neq 0)\cup (x_{t_{j}}\neq 0)\right)\right]}{\#\left[(x_{s_{j}}\neq 0)\cup (x_{t_{j}}\neq 0)\right]}\right)$$

(4) $${\mathrm{Braycurtis}}_{s,\;t}=\frac{\sum_{k=1}^{n}\left|x_{\mathrm{sk}}-x_{\mathrm{tk}}\right|}{\sum_{k=1}^{n}x_{\mathrm{sk}}+\sum_{k=1}^{n}x_{\mathrm{tk}}}$$

### Text Processing

#### Auto Encoder

**Figure 3.**
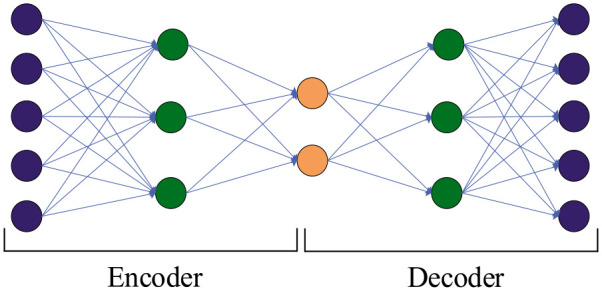
Structure of encoder and decoder.

The autoencoder is a neural network training process that reconstructs the input in low-dimensional vectors at the expense of an information loss. The autoencoder hidden layers have the effect of dimension reduction and provide expert natural language processing (Wei et al., 2020), which creates a hidden layer (or multiple hidden layers) in that a low-dimensional vector containing the meaning of the input data. Then, a decoder reconstructs the input data from the low-dimensional vectors through the hidden layers. Figure 3 presents the structure of the encoder and decoder.

The autoencoder is an unsupervised learning algorithm that only needs feed input data and does not need label or input/output paired data. The mean squared error (MSE) is adopted as the loss function. Equation (5) shows the calculation of the MSE loss function, in which y is the input data, $y^{,}$ is the output data of the autoencoder, and n is the sample size.

(5) $$\mathrm{Loss}(y,y^{,})=\frac{\sum_{i=1}^{n}{\left(y_{i}-{y_{i}}^{,}\right)}^{2}}{n}$$

#### Neural Network

A neural network with four hidden layers is constructed with TensorFlow, forming the encoding part of the autoencoder. In the decoding part, a neural network with four hidden layers corresponding to the coding part is also constructed. Based on previous research (Jung et al., 2019; Menaka & Vaidyanathan, 2022), we set the learning rate at 0.0001. The *n_batch* of each step is set at 7, the backpropagation amount of each epoch (*n_backpro*) is set at 300, and the attenuation coefficients *beta1* and *beta2* are set at .9 and .999, respectively. A 1080ti GPU provides computational power for the training of deep learning.

### Topic Analysis

#### Clustering Evaluation

Normalized mutual information (NMI) is used to evaluate the clustering effect (C. Xu et al., 2020), as it has significant advantages over other evaluation indicators. To be more specific, NMI is independent of the absolute values of the labels, which is the advantage of normalization. Therefore, the order of the levels will not change the score (Wickramasinghe et al., 2022). The calculation of mutual information depends on information entropy, which measures changes in information uncertainty. Equation (6) shows the calculation for information entropy.

(6) $$H(X)=-\sum_{i=1}^{n}p(x_{i})\log(p(x_{i}))$$

where H(X) is information entropy, n is the levels, and $p(x_{i})$ is the probability of event $x_{i}$ .

Mutual information is a practical measure in information theory. It can be regarded as the amount of information contained in a random variable or the uncertainty of a random variable, reduced by knowledge in another random variable (Akça & Yozgatlıgil, 2020). Equation (7) shows the calculation of mutual information.

(7) $$I(X;Y)=\sum_{x}\sum_{y}p(x,y)\log\frac{p(x,y)}{p(x)p(y)}$$

where I(X;Y) is the mutual information for X and Y , p(x,y) is the joint distribution of (X,Y) , and p(x)p(y) is the marginal distribution of (X,Y) .

NMI scales the mutual information in the interval [0,1], which makes it easy to evaluate and compare clusters. The higher the NMI, the more accurate the cluster is, and vice versa. Equation (8) shows the normalization method.

(8) $$\mathrm{NMI}(X;Y)=2\times \frac{I(X;Y)}{H(X)+H(Y)}$$

where NMI(X;Y) is the normalized mutual information for X and X , I(X;Y) is the mutual information for X and Y , H(X) and H(Y) are cross entropy for X and Y .

#### Topic Number

Distortion is typically used to measure the quality of clustering (Patel & Kushwaha, 2020). In a cluster, when the distortion is lower, the cluster members are closer, whereas when distortion is higher, the cluster structure is looser. However, the distortion decreases with an increase in the number of clusters. Therefore, we employ the Elbow method (Mouton et al., 2020) to estimate the relationship between the number of topics and distortions. Equation (9) shows the calculation for distortion, where *x* and *y* indicate cluster particles and sample points in clusters, respectively.

(9) $$d(x,y)={\left(x-y\right)}^{2}$$

**Figure 4.**
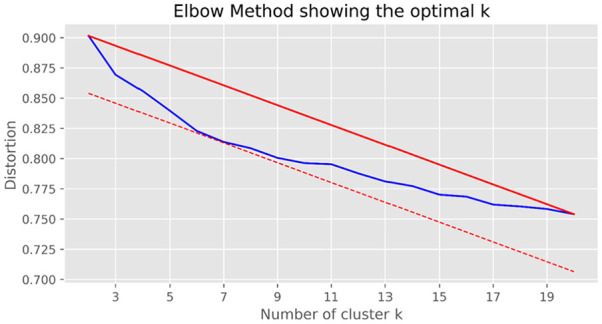
Distortion of the elbow method.

We calculate the distortion value in the interval [2, 20]. In Figure 4 the solid blue line represents the square distance error between the particle of each cluster and the sample point in the cluster, which is called the distortion; and the solid red line and dotted red line are the auxiliary lines for evaluating the degree of distortion. The degree of distortion decreases with an increase in categories, as the figure shows; but for data with a certain degree of differentiation, the degree of distortion will increase when a critical point is reached and then decline slowly. This critical point can be considered a point with better clustering performance and is a graphical representation of the optimal parameter. In Figure 4, the point that intersects the dotted line is 7, which can be regarded as an inflection point of the curve when the number of clusters is set at 7.

### Research Data

The research data come from Semantic Scholar (https://www.semanticscholar.org/cord19/), comprising COVID-19-related publications, known as CORD-19 (Ostaszewski et al., 2020). The dataset was created by the Allen Institute of Artificial Intelligence in collaboration with Zuckerberg, the Center for Security and Emerging Technologies at Georgetown University, Microsoft, IBM and the White House Office of Science and Technology Policy. It consists of more than 100,000 of these publications, some of which are not full texts and include only titles and abstracts. This free dataset is available to the global research community for the purpose of generating new insights to support the ongoing fight against the virus.

**Figure 5.**
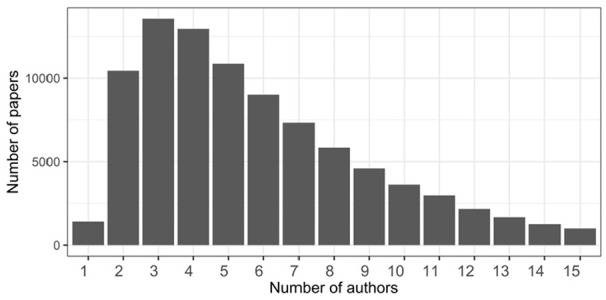
Statistics on number of papers and authors.

Figure 5 illustrates statistics on the number of papers and their authors. Most of the publications on COVID-19 have 2 to 10 authors, but only a few publications have only 1 author or more than 10 authors.

**Figure 6.**
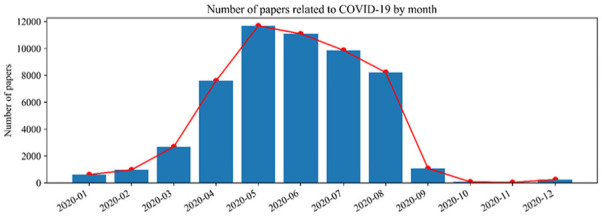
Number of publications in each month.

Cleaning and preprocessing of the data yield 54,136 COVID-19-related academic papers as the corpus for analysis. Because the outbreak of COVID-19 began in 2020, we retain only data starting in January 2020. Figure 6 gives a monthly breakdown of the number of publications: in January and February 2020 the number of publications was very low, but it grew rapidly in March and April; it reached a peak in May, when more than 10,000 papers on COVID-19 appeared. After September, the number of papers diminished because at that time, some papers had yet to be assigned to a volume and issue.

**Figure 7.**
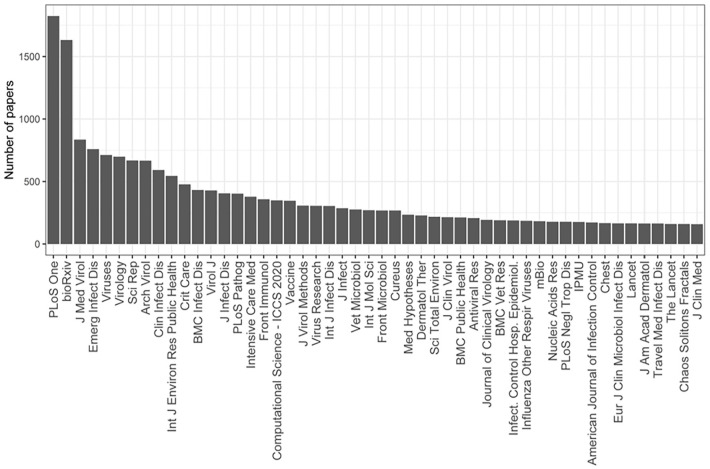
Papers in popular journals related to COVID-19.

Figure 7 shows the number of papers that appeared in the most popular journals related to COVID-19, showing that most of the research results about COVID-19 were published in multiple fields. *PLoS One* is the most popular journal publishing on COVID-19, indicating its sensitivity to emerging issues. In addition, *bioRxiv* also contains a large number of manuscripts on COVID-19. In general, publications related to COVID-19 take a medical focus.

## Results and Discussion

In this section, the results are presented with some discussions, including the result of distance comparison, topic extraction results, dynamics of clusters, and evolution in the future directions’ topics.

### Distance Comparison

**Figure 8.**
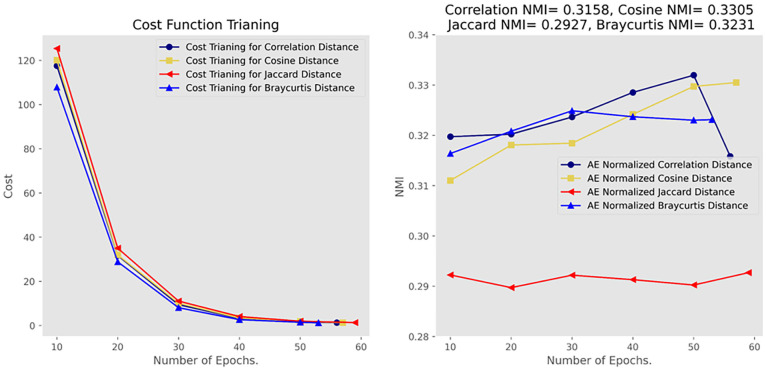
Comparison of distance functions. *Note*. NMI = normalized mutual information; AE = absolute entropy.

Figure 8 compares the distance functions. Based on the loss function, the four distance measurements converge after 50 to 60 epochs. Braycurtis converges the most quickly, and Jaccard has the slowest convergence. The cosine NMI has the highest distance measurements (0.3305) among the four, followed by Braycurtis, with an NMI of 0.3231 and correlation NMI of 0.3158. The Jaccard NMI ranks last, 0.2927. The results indicate that the clusters of the future directions achieve maximum discrimination when the cosine distance is adopted. Therefore, we adopt cosine distance for topic clustering.

### Topic Extraction Results

Table 1 lists the topics, representative keywords, and examples. Based on the cluster results, the projected research directions can be divided into the following seven topics: infectivity testing, genome analysis, vaccine testing, diagnosis and infection characteristics, pandemic management, nursing care, and clinical testing. By analyzing representative sentences, we can draw the following conclusions. First, many authors use predictive language in describing projected research, including “future studies,”“future research,”“future work,”“future steps,” and “future effects.” These words demonstrate a clear orientation toward the future. Second, most projected research is discussed from the perspective of medicine, including infection, gene analysis, vaccine testing, diagnosis, and viral analysis, and only a minority of it involves management, policy, government behavior, the economy, and so on. Discussion on COVID-19 from the perspective of management is a potential future research direction because some studies state that the pandemic had an fatal impact economically, psychologically, and on public administration (Nicola et al., 2020), but these fields have not been studied in depth in the existing research.

**Table 1.** Topics, Representative Keywords, and Examples.

Topic	Keywords	Examples
Infectivity testing	Infection	Future studies should include ……infection and severe …….
	Transmission	Future directions …… require new …… to mitigate disease transmission……
	Pathway	Future research will ……between insect and human cellular pathways……
Genome analysis	Genome	Future work will …… sheep genome sequences and ……
	Genotype	Future steps will be devoted to …… to genotype not only ……
	Sequence	We plan to carry out …… on the several thousand sequences for …….
Vaccine testing	Vaccine	In the future, it will be interesting to research on ……vaccine……
	Antibody	Further studies are needed to understand …… circulating serum antibody…….
	Dose-dependent	In our future work, we will take …… dose-dependent profiles……
Diagnosis and infection characteristics	Preventive	Hence, future efforts should be placed on …… preventive and ……
	Morbidity	Future studies on the comorbidity of ……
	Immunity	Future experimental …the role of autoimmunity ……
Pandemic management	Management	Future studies …… in the management of patients with …….
	Foundation	In a century’s time, we want future ophthalmologists to …… laid foundations for ……
	Program	Future improvements to…… in strategies for pathogen discovery programs ……
Nursing care	Care	Future research focused on …… to handle a surge in COVID-19 cases ……
	Symptom	Future studies should explore …… care in patients with serious COVID-19 with persistent symptoms ……
	Healthcare	Future studies should implement ……. improve the safety of our healthcare ……
Clinical testing	Assay	In the future, a more focused …… assay to study ……
	Serum	Future studies should determine …… serum chemistry changes ……
	Nucleic	A future perspective is …… the combination of nucleic acid with ……

### Dynamics of Clusters

**Figure 9.**
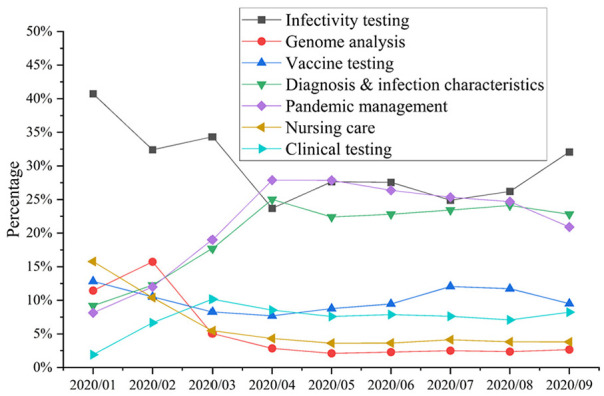
Dynamics of clusters (in 2020).

In the previous sections, we do not consider the time of publication. If we consider the time factor in topic clustering, that is, analyzing the future directions’ topics in the publications over time, then we can determine the dynamic changes in the topics, as shown in Figure 9.

Based on the dynamics of the cluster results, the topics pandemic management, diagnostic and infection characteristics, and clinical testing show an upward trend. Specifically, the proportion of mentions of pandemic management in papers increased from 8.15% in January to 20.91% in September, whereas the proportion of mentions of diagnosis and infection characteristics increased from 9.19% to 22.81%, and the proportion of mentions of clinical testing increased from 1.91% in January to 8.24% in September, demonstrating high growth. In other words, scholars’ expectations of the need for studying pandemic management, diagnosis and infection characteristics, and clinical testing are rising. On the one hand, this shows the value of these three topics. On the other hand, it shows that the problems faced in these three topic areas have not been solved so improvement is needed in dealing with them in the future.

However, some other topics that experienced a downward trend can be divided into two groups: (1) infectivity testing, a topic whose share of mentions is more than 20%; and (2) genome analysis and nursing care, whose share is less than 20%. Finally, in the observation window, the share of mentions of vaccine testing neither rises nor declines, but remains at around the same level.

Notably, the only topic that comes the perspective of government function, public administration, and economic development, rather than medicine, is pandemic management. This topic has a share of about 20%, that is, about one-fifth of the publications intend to expand into the field of management in the future, but about 80% of the future directions are still related to medicine.

### Evolution in the Future Directions’ Topics

**Figure 10.**
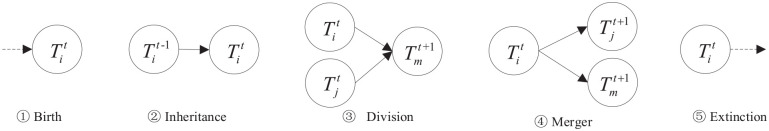
Types of topic evolution. *Note. T* = text corpus; *t* = time parameter of phase *t; i* = *i*th topic; *i* = *j*th topic.

Using the time factor, we can deeply analyze the process of evolution in the topics of scholars’ future research, that is, the topic life cycle between emergence and extinction over a certain period. Based on the existing research, we divide the types of topic evolution into five categories that correspond to the types of topic evolution: birth, inheritance, division, merger, and extinction (Qin & Le, 2015), as shown in Figure 10.

**Figure 11.**
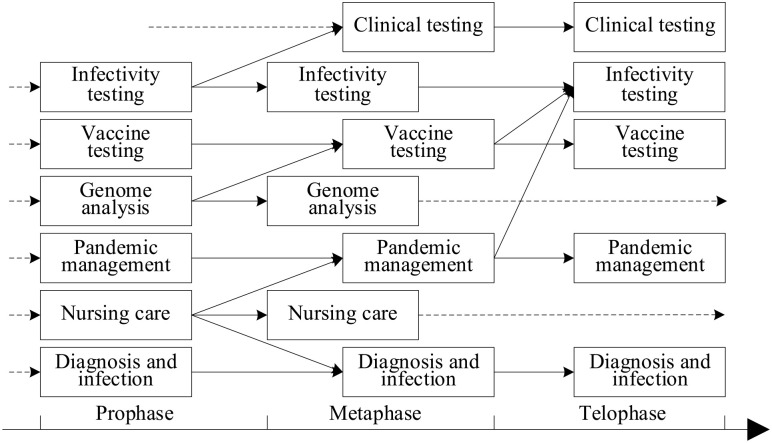
Evolution of future directions’ topics.

We regard 5% as the threshold between birth and extinction (i.e., if the proportion is more than 5%, it is considered a birth; otherwise, it is considered extinct; Ridley et al., 2007). The purpose of this division is to investigate the evolutionary trend in COVID-19 research topics, which is the focus of researchers in the research cycle. If the threshold is too large, then the change is likely to be ignored, but if it is too small, then changes will easily attract a lot of attention; in practice, 0.05 is a commonly used threshold parameter (Ridley et al., 2007). Then, we divide the period into three stages: prophase, metaphase, and telophase of the topic: prophase is the rising stage, metaphase is the growth stage, and telophase is the degradation stage. Based on these five types of topic evolution, we measure the evolution process of different topics, as shown in Figure 11.

Because the threshold is 5%, and the proportion of topics on clinical testing in the first month is 1.91%, we determine that the topic clinical testing began in the metaphase stage and in part grew out of infectivity testing, that is, the topic of effectiveness testing split, and the result of one split is clinical testing. However, the topics of pandemic management and diagnosis and infection characteristics obtained part of the content from the topic nursing care after its emergence—that is, what was expressed as nursing care at first was later absorbed into pandemic management and diagnosis and infection characteristics. Moreover, what was first called pandemic management and diagnosis and infection characteristics are retained in the follow-up periods. In addition, the topic vaccine testing grew in part from the integration of vaccine testing and genome analysis in the metaphase, but, in the telophase stage, some content was absorbed by the infectivity testing topic. In the telophase stage, genome analysis and nursing care withered away.

## Conclusion and Prospects for Future Research

In this study, we focus on the future directions of COVID-19-related publications and collected data from Semantic Scholars to carry out text mining. After data cleaning and preprocessing, 54,136 COVID-19-related academic publications were selected as the corpus, and we extracted the future directions of the publications as the data source for text analysis. Then, an autoencoder model was built to cluster topics, and the Elbow method was employed to identify the optimal number of clusters. The results show that the future directions’ topics in COVID-19-related publications can be divided into seven clusters, and cosine distance can obtain the maximum NMI. The seven clusters are infectivity testing, genome analysis, vaccine testing, diagnosis and infection characteristics, pandemic management, nursing care, and clinical testing. Pandemic management, diagnosis and infection characteristics, and clinical testing experienced rapid growth, vaccine testing remained steady in the observation window, and other topics (infectivity testing, genome analysis, and nursing care) slowly declined. This study enriches our understanding of COVID-19, expands text value mining in the field of COVID-19, and provides guidance for the operation of management measures in practice.

However, the study has some shortcomings, which could be address by future research in the following ways. First, in this study, we use unsupervised clustering to process the future directions. An unsupervised clustering algorithm does not automatically identify the best cluster, and manual intervention is needed to determine the number of clusters. Although we used the Elbow method to assist in this evaluation, it is undeniable that individual judgment still has a decisive impact. In the future, we plan to perform supervised classification of the future directions, that is, to classify documents on the basis of designated topics to obtain more consistent results with human perception. Second, the narrative is domain dependent, leading the future directions on COVID-19 involve many medical domain-specific terms, such as *coronavirus*, *SARS-CoV-2*, and *immunocompromised*. Although we used the special package (*Scispacy*) for mining medical text, the package lags behind the rapid evolution in COVID-19 research. For more accurate modeling in follow-up research, we plan to introduce field terminology knowledge specific to COVID-19. Third, we extracted the text of the future directions from publications as the research corpus, but some publications do not provide future directions. A complete investigation of the publications is also a direction for future research, for example, including the title, abstract, and conclusion in the manuscripts. Fourth, the research corpus comprises only English-language publications, so publications in other language are not included. Future research could consider a corpus in other languages. Finally, we omitted publications with missing values from the corpus, for example, some papers provide only the author names, titles, and abstracts but lack full-text content, but these publications should not be overlooked, forming another direction for future research.
